# Screening of antibiotic residue in transported live fish and water collected from different fish markets in Mymensingh district of Bangladesh

**DOI:** 10.5455/javar.2022.i574

**Published:** 2022-03-10

**Authors:** Md. Mehedi Hasan, Kazi Rafiq, Most. Rifat Ara Ferdous, Md. Tarek Hossain, Arifa Parvin Ripa, Shahroz Mahean Haque

**Affiliations:** 1 Department of Fisheries Management, Bangladesh Agriculture University, Mymensingh, Bangladesh; 2 Department of Pharmacology, Bangladesh Agriculture University, Mymensingh, Bangladesh; † These two authors contributed equally to this work.

**Keywords:** Catla, Rui, Mrigal, Pangas, HPLC, oxytetracycline

## Abstract

**Objectives:**

The objective of this study was to evaluate the persistence of oxytetracycline (OTC) residue in common fish species (Catla, Rui, Mrigal, and Pangas) available in local fish markets and the corresponding transport water of the Mymensingh region.

**Materials and Methods:**

Live fish and corresponding transport water samples were analyzed by thin layer chromatography (TLC) and high-performance liquid chromatography for qualitative and quantitative detection of OTC residue, respectively. A total of 240 fish samples and 60 water samples were randomly collected from three local fish markets during the summer and winter seasons.

**Results:**

OTC residues were detected in 18 samples (13 fish and 5 water samples) in the summer and 8 samples (only fish samples) in the winter. The overall percentage of antibiotic residue positive in fish samples was 5.42%, and in water samples, it was 8.33%. In fish, OTC concentrations of TLC-positive samples ranged from 34.7 to 56.85 parts per billion (ppb) in Catla, 23.45–35.37 in Rui, 11.02–26.80 in Mrigal, and 10.80–77.55 in Pangas during summer. The concentrations were 18.5 ppb in Catla, 15–16.09 in Rui, 10–14.63 in Mrigal, and 21.02–40.11 in Pangas during the winter season. On the other hand, the range of OTC concentrations of TLC-positive samples for water was 12.9–59.18 ppb during summer and below the detection level during winter. The highest prevalence of antibiotic residues among fish samples was found in Pangas (16.67%). The highest percentage of samples (15% in the fish sample and 30% in the water sample) found to be positive were collected from Mechua Bazar. The comparison between the summer and winter seasons showed that the percentage of positive antibiotic residue in the summer season (10.38% for fish and 16.67% for water) is higher than that of the winter season (6.67% for fish samples only). This variation indicates that fish transporters use more antibiotics during the summer than in the winter season. The difference between the means of fish species and water samples was not statistically significant (p > 0.05). In addition, no samples exceeded the maximum residue limits (MRL) of OTC (100 ppb) in fish set by the European Commission.

**Conclusion:**

Although the concentrations of antibiotic residues in fish edible tissues are below MRL values, the presence of antibiotic residues in transported water may lead to the development of antimicrobial resistance bacteria that are detrimental to humans, animals, and aquatic animals.

## Introduction

Bangladesh has a diverse range of fishing resources, which can be divided into two categories: inland fisheries and marine fisheries. Inland fisheries occupy 47.60 lakh MT and are divided into two subsectors: inland catch and inland culture [1]. Fisheries and aquaculture are two of the most productive and developing industries in Bangladesh’s economy, with enormous potential for future growth. The fishing sector can directly contribute to pro-poor aims by providing jobs and a source of income. The total fishing sector directly or indirectly supports the livelihoods of about 18 million people in the country [1]. Approximately 1.4 million women in the fishing, farming, fish handling, and processing industries depend on their jobs for money [2].

According to a recent analysis [3], fish is the predominant protein source in the Bangladeshi diet, accounting for around 60% of total animal protein. Per capita fish consumption in the country is 62.58 gm, which is greater than the daily protein demand (60 gm). Fisheries have traditionally been important to the national economy as a key source of animal protein, employment opportunities, nutrition security, foreign revenues, and socioeconomic development in an agro-based society [1]. It generates 3.61% of the national GDP of Bangladesh and roughly 24.41% of the agricultural GDP [4]. This sector’s average growth rate over the last 10 years has been around 5.43%. In 2018, Bangladesh ranked 3rd in inland fish production, 5th in aquaculture production, and 11th in marine fish production [5]. Bangladesh is currently self-sufficient in fish production and has begun to gain international prominence as one of the world’s largest fish producers [1]. As a result, fish and fisheries have long been an important part of the Bangladeshi people, and they continue to play an important role in providing nutritional needs, creating jobs, earning foreign currency, and other sectors of the economy [6].

Rui, Catla, Mrigal, and other large Indian carps are among the most popular fish in Bangladesh. Like other cultured catfish, Pangas are well known among fish farmers for their fast growth, simple culture system, robust features, high survival rate, capacity to survive at high stocking densities, strong disease resistance, and tolerance of a wide range of environmental factors [7]. Recently, people have used drugs and chemicals during transportation for several reasons, such as disease treatment, reducing metabolic rates, reducing excitability of fish, and convenience in handling fish. Different types of therapeutic agents and aquatic chemicals are used to treat affected aquatic animals [8]. Different types of antibiotics are also used for disease treatment purposes [9]. In aquaculture systems, oxytetracycline (OTC) is one of the most popular antibacterial agents for disease treatment purposes, control of diseases, and an in-feed growth promoter [10]. Because of their broad spectrum of activities and low cost with easy availability, today’s OTC antibiotics are abundantly used in fish farms as prophylactic in fresh water aquaculture in Bangladesh [11]. However, antibiotics cannot be used correctly during disease treatment in aquaculture practices [12]. Indiscriminate use of antibiotics could lead to undesirable deposition of their residues in edible tissues of fish and could be hazardous for public health [13]. Antibiotic residues transferred to humans through the food chain may also alter the intestinal ecology, thereby favoring the emergence of resistant microflora [14]. Therefore, the consumption of aquatic food containing antibiotic residues is a global concern. In addition, about 70%–80% of drugs used in aquaculture end up in the environment, which can be transferred to human beings through food contact with the fish or water [15]. Antimicrobial residues in aquatic food also result in lower marketing and export values of aquaculture products [16,17]. Therefore, special emphasis should be given to limiting antibiotic use and residue contamination in the fishing sector because of its potential hazards to public health and combating antimicrobial resistance (AMR) for safe aquatic food production.

However, in Bangladesh, indiscriminate use of antibiotics in fish culture has been reported by several authors and the quantitative assessment of antibiotic residues in fish is limited to transporting live fish and water. Therefore, our present study was designed to investigate the qualitative and quantitative determination of OTC residues in transported live fish and corresponding transport water in different fish markets in the Mymensingh district of Bangladesh.

## Materials and Methods

### Ethical approval

The experiment was carried out in the Department of Pharmacology at Bangladesh Agricultural University, Mymensingh. All experimental procedures were conducted in accordance with the guidelines for the care and use of animals as established by the Animal Welfare and Experimentation Ethics Committee (AWEEC) of Bangladesh Agricultural University, Mymensingh [Approval number: AWEEC/BAU/2018(11)].

### Study area and collection of ﬁsh samples

The present study was conducted in three different fish markets in Mymensingh to collect fish and water samples transported from other local fish markets in Bangladesh, i.e., Rajshahi, Natore, Jessore, Sylhet, Khulna, etc. These fish markets were Mechua Bazar, Shankipara Bazar, and Bypass Mor Bazaar. Four different fish species such as Catla (*Catla catla*), Rui (*Labeo rohita*), Mrigal (*Cirhinus *cirrhosus), and Pangas (*Pangasius pangasius*) available in local fish markets of Mymensingh upazilla were selected to detect the persistence of OTC residue. A total of 120 fish samples (40 samples from each market) and 30 water samples (10 samples from each market) in the summer season (May to July 2019) and 120 fish samples (40 samples from each market) and 30 water samples (10 samples from each market) in the winter season (November 2019 to January 2020) were randomly collected from three local fish markets in the study area. The fish samples were collected in separate polythene bags with proper tagging from the several fish markets and maintained in an ice box with enough ice. Fish and water samples were collected and brought to the Department of Pharmacology, Bangladesh Agricultural University. They were kept in a deep refrigerator (–18°C) for further processing and analysis to detect OTC residue.

### Screening of residue by thin layer chromatography (TLC)

#### TLC apparatus

A locally prepared TLC plate (0.25 mm thickness, MN, Germany), a locally prepared TLC chamber, and a UV detection box will be used to detect the antimicrobial residue from samples.

#### Preparation of the standard

OTC was collected from Merck, Germany. The OTC standard was prepared by dissolving 0.1 gm of antibiotic powder into 4 ml of methanol solution as described previously [18].

#### Preparation of fish tissue for analysis of antibiotic residue

The sample extraction procedure was carried out according to Sattar et al. [19] with some modifications. About 2 mg of fish muscle was taken in a falcon tube for TLC. Then, the taken sample was cut into small pieces, ground, and blended. For homogenization, 10 ml of phosphate buffer saline (pH 6.5) was added to the samples and vortexed for 1 min. After homogenization, 2 ml of 30% trichloroacetic acid was added to the sample and shaken immediately for protein precipitation, then centrifuged at 6,000 rpm for 15 min. The supernatant was collected and filtered by Whatman 125 mm filter paper and funnel in another properly cleaned falcon tube. At least 2 ml of the supernatant was taken, and the same amount of diethyl ether was added, followed by an 8–10 min wait at room temperature. 1 ml was taken from the bottom of the falcon tube and then placed into a TLC plate, and then the plates were placed on the TLC tank, which contained the mobile phase. After placing the plates, the TLC tank was covered by a lid and left until the mobile phase reached the upper line. Then, the plates were dried and 256 nm wave length UV light was used to visualize the residue spot in the stationary phase in the UV detection box. The spot was marked with a pencil to calculate the retention factor (*R_f_*). *R_f_* values were calculated by measuring the distance traveled by the solvent and the distance traveled by individual spots. A compound with the same *R_f_* value as the standard is considered comparable [20].

#### Quantitative determination of antimicrobial drugs residue by high-performance liquid chromatography (HPLC)

Quantification of antibiotic residue was carried out by HPLC test. 

#### UHPLC apparatus

Thermo Scientific UltiMate 3000 Autosampler Column Compartment UHPLC from Waltham, MA, was used. Synchronys C18 reverse-phase stainless steel column (carbon load 16%, 250 mm length, 4.6 mm diameter, 100 Å (10 nm) pore size, 5 µm particle size, and surface area m^2^/g) from Thermo Scientific, Waltham, MA, was used as the stationary phase. For sample preparation, a refrigerator, a centrifuge machine (Tabletop Centrifuge, DSC-200A-2, Taiwan), an ultrasonic bath (ISOLAB Laborgerate GmbH, Germany), a filter machine (Rocker 300, Taiwan), a rotary evaporator (IKA-Werke GmbH and Co., Germany), a homogenizer (Mini Vortex Mixer, VM-100-B, Taiwan), and a 0.2 MFS syringe filter (Advantec MFD, Japan) were used.

#### Preparation of standard

Each antibiotic’s primary standard stock solution was prepared by dissolving 10 mg of antibiotics into 10 mg of mobile phase (different for each antibiotic) to give a final concentration of 1 mg/ml. The stock solution was kept in amber glass vials to prevent photo-degradation and stored at −20°C in the refrigerator. The stock solution was used within 4 weeks of preparation [21]. The secondary standard solution of each antibiotic was prepared by following the maximum residue limit (MRL) values prescribed by the Codex Alimentarius Commission of the World Health Organization (WHO) [22]. Based on the MRL values, a linearity range (½, 1, 2, 4, and 6 times the MRL value) was selected to cover the lowest MRL values for OCT. Then, the primary standard solution was diluted with the mobile phase to the required volume to prepare the secondary standard solution [23].

#### Preparation of samples

About 2 gm/2 ml of the five blank samples (free from antibiotics) were spiked with 100 µl of five secondary standard solution, followed by thorough mixing, and allowed to stand for 15 min [24]. All the fortified samples and unknown samples were subjected to an extraction and cleanup procedure as described in TLC. Before use for HPLC, the required amount (20 µl) from each sample was filtered through a 0.2 syringe filter (Advantec MFD, Japan).

#### Fortification of samples

Fortification was done to study the linearity as well as recovery rates. About 2 gm/2 ml of the five blank samples (free from antibiotics) were spiked with 100 µl of five secondary standard solutions, followed by thorough mixing, and allowed to stand for 15 min.

#### UHPLC procedure

Samples were run in the UHPLC machine according to the previously published procedure [25] with some modifications.

#### Recovery evaluation

Recovery rate was calculated based on the following equation [26]: 


$$\%\mathrm{Recovery}=\frac{\mathrm{Concentration}\;\mathrm{of}\;\mathrm{the}\;\mathrm{spiked}\;\mathrm{sample}–\mathrm{Concentration}\;\mathrm{of}\;\mathrm{unspiked}\;\mathrm{sample}}{\mathrm{Concentration}\;\mathrm{of}\;\mathrm{added}\;\mathrm{antibiotics}\;\mathrm{in}\;\mathrm{the}\;\mathrm{spiked}\;\mathrm{sample}}$$

#### Preparation of calibration curve

**Figure 1.**
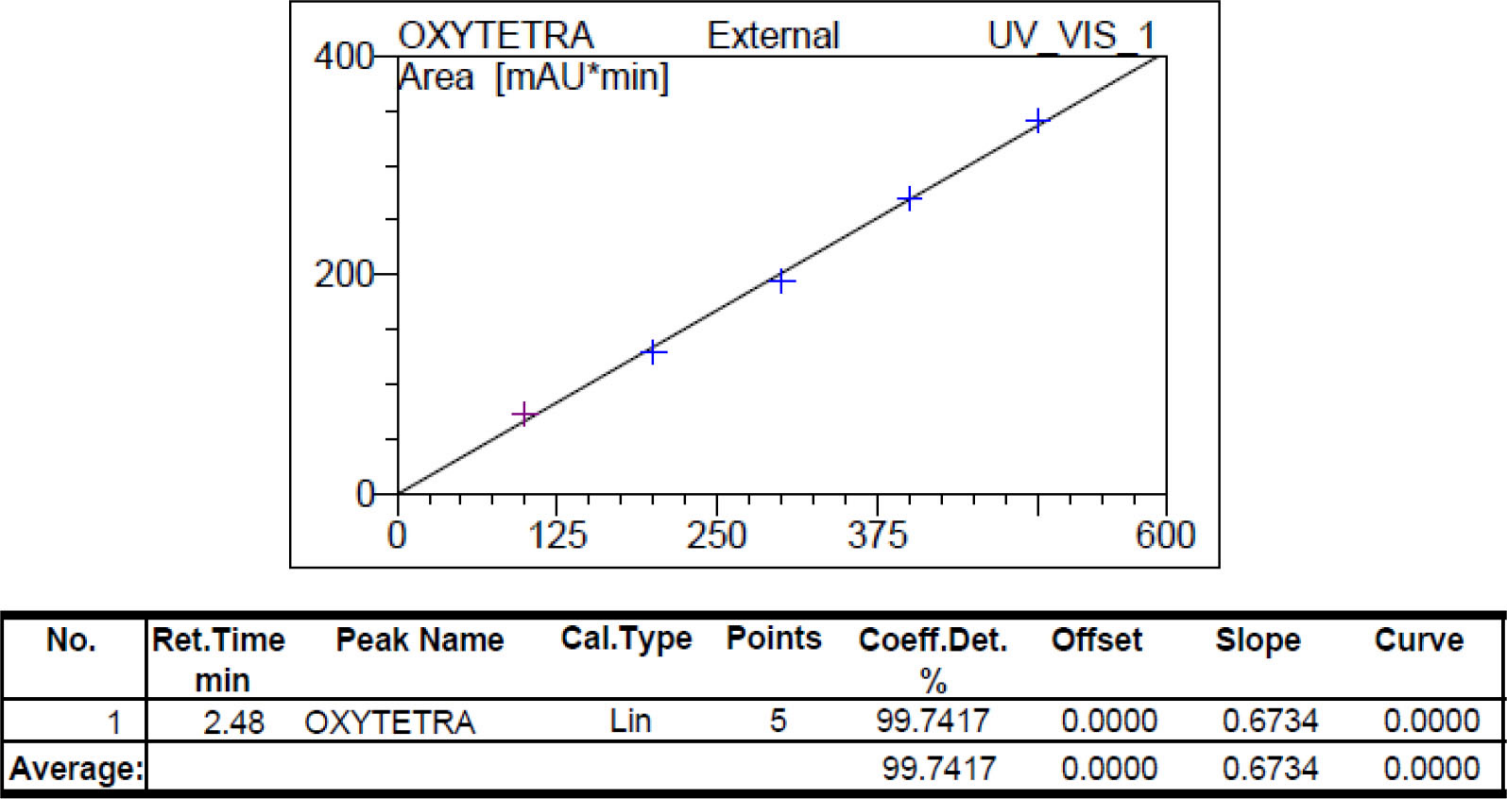
Calibration curve for OTC during HPLC analysis.

Calibration curve was prepared from injecting corresponding concentrations of OTC standard solutions of 0, 125, 250, 375, 500, and 600 parts per billion (ppb). The linear fit curve was obtained by using the following equation:

*y = mx + b*;

= 0.0132368*x*+ 0.04568

where *y* = peak area, *x* = concentration of OTC (ppb), and the correlation coefficient (*r*²) = 0.99687. The mean retention times of the OTC was found to be 2.48 min (Fig. 1).

#### Statistical analysis

GraphPad Prism Statistical Software version 8 was used to analyze the data (GraphPad Software, San Diego, CA, www.graphpad.com). When comparing the mean values of two variables, an unpaired *t*-test was carried out, and when comparing the mean values of more than two variables, a one-way analysis of variance, followed by a *post-hoc* test, was employed. The significant alpha value was chosen at *p* ≤ 0.05. 

## Results and Discussion

The present study aimed to assess the prevalence of antibiotic residue in the transported live fish and corresponding transport water samples from three different fish markets (Mechua Bazar, Shankipara Bazar, and Bypass Mor Bazar) in the Mymensingh region during different seasons. The prevalence of antibiotic residue observed in different samples varies with the sample type and seasons and is discussed below.

In the present study, OTC residue was detected in 02 (6.67%) Catla, 03 (10%) Rui, 03 (10%) Mrigal, and 05 (16.67%) Pangas samples, as well as 05 (16.67%) transport water samples during the summer season (May–July/2019). The range of detected OTC concentrations of TLC-positive samples (Table 1) was 34.7–56.85 ppb (mean = 45.78 ± 11.08 ppb) in Catla, 23.45–35.37 ppb (mean = 29.26 ± 3.44) in Rui, 11.02–26.80 ppb (mean = 17.24 ± 4.85) in Mrigal, and 10.80–77.55 ppb (mean = 42.94 ± 12.31) in Pangas by HPLC. OTC residues in 107 (89.17%) fish samples were found to be less than the detection limit under the study period. In our current study, 13 (10.83%) fish samples were found positive through the TLC plate method.

As shown in Table 2, the total amount of OTC residue was detected in 5 (16.67%) water samples. The range of detected OTC concentrations of positive samples was 12.9–59.18 ppb (mean = 32.24 ± 8.91 ppb). However, OTC residue in 25 (83.33%) water samples was less than the detection level.

**Figure 2.**
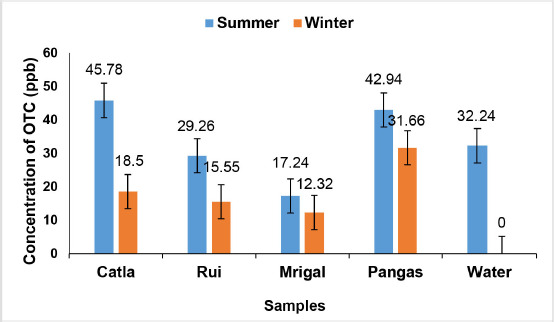
Comparison of detected OTC residue in Catla (*C. catla*), Rui (*L. rohita*), Mrigal (*C. cirrhosus*), and Pangas (*P. pangasius*) between the summer and winter seasons.

During the winter season (November–January), OTC residue was detected in 01 Catla (3.33%), 02 Rui (6.67%), 02 Mrigal (6.67%), and 03 Pangas (10%) samples. The total amount of detected OTC residue of TLC-positive samples (Table 3) was 18.5 ppb (mean = 18.5 ppb) in Catla, 15–16.09 ppb (mean = 15.55 ± 0.545) in Rui, 10–14.63 ppb (mean = 12.32 ± 2.315) in Mrigal, and 21.02–40.11 ppb (mean = 31.66 ± 5.62) in Pangas. In 112 (93.33%) fish samples, OTC residue was found to be less than the detection level. In the present study, the range of OTC residue was found in eight (6.67%) fish samples, which was below the detection limit compared with the MRL of OTC (100 ppb) set by the European Commission (Fig. 2). 

Several previously published reports also detected OTC residue in fish. OTC residue was found in 13 salmon fish samples from farms [27]. Another study also found that OTC residue in cultured fishes collected from the coast of Korea and their natural habitat was up to 60 ppb [28]. In agreement with our current data, an earlier report showed that OTC residue was detected in 05 (20.83%) Tilapia *(Oreochromis niloticus)*, 09 (37.50%) Thai Koi, and 06 (25%) Pangas fish samples [29]. OTC concentrations of positive samples were also found by Barman et al. [30] and were 23.77–39.94 ppb (mean = 38.88 ± 2.99 ppb) in Tilapia *(O. niloticus)* and 29.61–55.98 ppb (mean = 42.3 ± 3.00 ppb) in Thai Koi. Research was conducted on 50 rainbow trout muscles to evaluate the OTC residues. They were collected from the different fish markets in Pakistan, where the residue was below the detection level [31]. A study was conducted in Shahre-Kord, Iran, in which OTC residue was also found in 03 (6%) of the samples before frying and 24% (12) of the samples after frying in rainbow trout *(Oncorhynchus mykiss)* meat. They also reported that 63.1% of the samples contained tetracycline residue in rainbow trout meat, where maximum samples were under the detection level and only one sample (101.40 ppb) exceeded the MRL of OTC set by the European Commission [32]. Research was conducted on 70 fish samples from 70 different fish farms in Mugla province of Turkey where they did not find any tetracycline residue (OTC, tetracycline, chlortetracycline, and doxycycline) that crossed the detection limit [33]. The result is also related to some findings where OTC residue was not found in any samples of fish (*Oblada melanura* and *Mullus barbatus*) [34]. Another research also demonstrated that tetracycline residue in O. mykiss was 8.44 ± 6.03 ppb, which was lower than the detection limit [35]. OTC residue in three fish samples hunted from surrounding fish farms in Muğla district exceeded the MRL laid down in the Codex (100 ppb) [36]. In addition, a survey was conducted in Nigeria that reported OTC residue in 30% of the fillet samples of 160 catfish collected from different fish farms and restaurants contaminated with OTC residue, and the range was 22.5–553.2 ppb (18.8%), which exceeded the limit of 200 ppb set by the Codex Alimentarius Commission [37].

**Table 1.** Presence of antibiotic residue (OTC) in fish samples during the summer season.

Fish samples	Total samples number	Positive sample number	Concentration (ppb, µg/kg)		Recovery %	*R* ^2^	Exceed MRL^a^ *n*, (%)
			Mean ± SEM	Range			
Catla	30	02	45.78 ± 11.08	34.7–56.85	85	0.997	0, (0.00)
Rui	30	03	29.26 ± 3.44	23.45–35.37			0, (0.00)
Mrigal	30	03	17.24 ± 4.85	11.02–26.80			0, (0.00)
Pangas	30	05	42.94 ± 12.31	10.80–77.55			0, (0.00)

a MRL = 100 ppb [Food and Drugs Administration (FDA)], 200 ppb (FAO/WHO).

**Table 2.** Presence of antibiotic residue (OTC) in water samples during the summer season.

Total water samples number	Positive samples number	Concentration (ppb)		Recovery %	*R* ^2^	Exceed MRL^a^ *n*, (%)
		Mean ± SEM	Range			
30	05	32.24 ± 8.91	12.9–59.18	85	0.997	0, (0.00)

a MRL = 100 ppb (FDA), 200 ppb (FAO/WHO).

**Table 3.** Presence of antibiotic residue (OTC) in fish samples during the winter season.

Fish samples	Total samples number	Positive sample number	Concentration (ppb)		Recovery %	*R* ^2^	Exceed MRL^a^ *n*, (%)
			Mean ± SEM	Range			
Catla	30	01	18.5	18.5	85	0.997	0, (0.00)
Rui	30	02	15.55 ± 0.545	15–16.09			0, (0.00)
Mrigal	30	02	12.32 ± 2.315	10–14.63			0, (0.00)
Pangas	30	03	31.66 ± 5.62	21.02–40.11			0, (0.00)

a MRL = 100 ppb (FDA), 200 ppb (FAO/WHO).

**Table 4.** Presence of antibiotic residue (OTC) in water samples during the winter season.

Total water samples number	Positive samples number	Concentration (ppb)		Recovery %	*R* ^2^	Exceed MRL^a^ *n*, (%)
		Mean ± SEM	Range			
30	00	BDL	BDL	85	0.997	BDL

a BDL: Below detection level.

It is important to note that many researchers have detected the presence of antibiotics. However, to the best of our knowledge, there is no data yet published regarding the antibiotic residues during seasonal variation in both transported live fish and water collected from different fish markets in the Mymensingh district of Bangladesh. In the current study, the comparison between the summer and winter seasons showed that the percentage of positive antibiotic residue in the summer season (10.38% for fish and 16.67% for water) is higher than in the winter season (6.67% for fish and 0% for water). In addition, no residue is present in the transported water during the winter session. This variation indicates that fish transporters use more antibiotics in the summer season than in the winter session (Table 4). In addition, OTC residue in water samples was found more in the summer than in the winter season. One reason for this may be that the water is changed more frequently in the winter than in the summer during transportation of fish, and also due to low disease prevalence and mortality, as well as heat stress. In the current study, the presence of OTC in fish and water speculates that the sources of antibiotic residues may come from the use of antibiotics in fish feed used to prevent disease or for prophylactic purposes during fish farming. On the other hand, in both summer and winter seasons, OTC residue was found mostly in Pangas rather than Catla, Rui, and Mrigal. These results are in agreement with the previous finding where they reported that six Pangas fish samples were contaminated with OTC residue [29]. A previous study also reported that the most commonly used antibiotic is OTC and was found in Koi and Pangas fish species. It is the most commonly used antibiotic compound compared to the others [11]. Smith et al. [38] also reported that OTC is one of the most widely used antibacterials in aquaculture worldwide and that the vast majority of OTC supplied in supplementary feed can be found in hatchery effluent [38]. Therefore, it can be said that antibiotics may enter into the fish body not only through mixing with water during transportation but also through antibiotics mixed as supplementary growth promoters in the feed of fish during the farming system. 

The present study found that the overall percentage of antibiotic residue in fish was not statistically significant and was below the MRL values. These data suggest that the residual level may not harm fish health or be bad for humans to eat. However, it can cause two different problems. Firstly, it can cause acute toxicity, which may lead to diarrhea, vomiting, nausea, cancer, and problems in the human body’s digestive system. In addition, low levels of antibiotic residues also alter the normal gut microfloral environment in both human and fish gut, leading to abnormal digestion or poor digestibility. Secondly, it may cause bacterial resistance that will kill the beneficial bacteria in the human, animal, and fish bodies. In addition, OTC residue could contact the human body via the food chain [39]. On the other hand, antibiotic residue in fish and around fish farms could make fish more resistant to antibiotics, which could then spread to humans and animals, which could have bad effects on AMR [40,41]. 

Nowadays, food safety has become an alarming issue worldwide. As it is a profitable business in Bangladesh, a wide range of people are directly or indirectly involved in fish and fishing-related occupations. However, the majority of them are unfamiliar with antibiotics and their use in fish culture for disease treatment and prevention. Earlier, it can be seen that this practice had an influence on fish as well as on humans. Furthermore, the indiscriminate use of antibiotics in fish culture may result in undesired drug residue deposits in the edible tissues of fish muscle, posing a public health concern to consumers.

From the above discussion, it can be easily understood that indiscriminate use of antibiotics bears no beneficial outcome. On the other hand, the market value of aquaculture products may decrease due to the presence of antibiotic residues [42,43]. In this case, there must be a need to take some corrective actions to ensure the safety of contaminated with antibiotic-free fish for consumers. Therefore, OTC use at field level must be kept under supervision and can be used in fish culture with prescribed doses only for disease treatment purposes. The government must implement some measures targeted at reducing the need for antibiotics during aquaculture practices and transportation purposes under a safe limit and ensure their prudent use. Furthermore, the isolation and identification of antibiotic resistance bacteria in fish and water of fish farming areas must be monitored, and antibiotic residue levels must be observed in other fish species.

## Conclusion

OTC residues are present in a small portion of fish species in the Mymensingh region of Bangladesh. Although OTC residues in fish species and water samples in both summer and winter seasons did not exceed the MRL recommended by the European Commission, it must be needed to monitor the indiscriminate use of antibiotics in fish culture for the consumer. In addition, some corrective and preventative measures are also required to assure drug residue-free, safe fish production for human consumption. Regular monitoring of marketed fish should be carried out by government authorities, and raising public awareness must be needed to provide a safe and healthy life, as well as to combat AMR. 

## List of Abbreviations

AMR, Antimicrobial resistance; AWEEC, Animal Welfare and Experimentation Ethics Committee; BDL, Below detection level; FDA, Food and Drugs Administration; HPLC, High-performance liquid chromatography; UHPLC, Ultra-High Performance Chromatography; FAO, Food and Agriculture Organization; OTC, Oxytetracycline; ppb, Parts per billion; MRL, Maximum residue limits; TLC, Thin layer chromatography; WHO, World Health Organization.

## References

[1] Fisheries Resources Survey System, FRSS. (2017). Fisheries statistical report of Bangladesh, Department of Fisheries, Dhaka, Bangladesh. Fisheries Resources Survey System.

[2] Bangladesh Foreign Trade Institute, BFTI. (2016). Study on sector-based need assessment of business promotion council—fisheries products Kawran Bazar, Dhaka, Bangladesh.

[3] Statistical yearbook of Bangladesh, BBS. (2017). Bangladesh bureau of statistics.

[4] (2017). DoF Annual report.

[5] FAO (2018). The state of world fisheries and aquaculture (opportunities and challenges).

[6] Alam MF, Penman DJ, Hussain MG, McAndrew BJ, Mazid MA (2002). Socio-economic aspects of carp production and consumption in Bangladesh.

[7] Begum M, Akter T, Minar MH (2012). Effect of salt and garlic on the quality and microbial content of smoked catfish (*Pangasianodon hypophthalmus*). Int J Bio Res Stress Manag.

[8] Rico A, Phu T, Satapornvanit K, Min J, Shahabuddin AM, Henriksson PJG (2013). Use of veterinary medicines, feed additives and probiotics in four major internationally traded aquaculture species farmed in Asia. Aquaculture.

[9] Avsever ML, Türk N, Tunaligil S (2010). The increase of antibiotic resistance in aquaculture and its effects on human health. Born Vet Kontr Araşt Enst.

[10] Erdogdu AT (2012). Using antibiotics in aquatic living beings.

[11] Ali H, Rico A, Murshed-e-Jahan K, Belton B (2016). An assessment of chemical and biological product use in aquaculture in Bangladesh. Aquaculture.

[12] FAO/WHO (2003). Code of practice for fish and fishery products.

[13] Cabello FC (2006). Heavy use of prophylactic antibiotics in aquaculture: a growing problem for human and animal health and for the environment. Environ Microbiol.

[14] Perrin-Guyomard A, Cottin S, Corpet DE, Boisseau J, Poul JM (2001). Evaluation of residual and therapeutic doses of tetracycline in the human-flora-associated (HFA) mice model. Regul Toxicol Pharmacol.

[15] Serrano HP (2005). Responsible use of antimicrobials in aquaculture. FAO Fish Tech Paper.

[16] Heuer OE, Kruse H, Grave K, Collignon P, Karunasagar I, Angulo FJ (2009). Human health consequences of use of antimicrobial agents in aquaculture. Clin Infect Dis.

[17] Sapkota A, Sapkota AR, Kucharski M, Burke J, McKenzie S, Walker P (2008). Aquaculture practices and potential human health risks: current knowledge and future priorities. Environ Int.

[18] Ferdous MRA, Ahmed MR, Khan SH, Mukta MA, Anika TT, Hossain MT (2020). Effect of discriminate and indiscriminate use of oxytetracycline on residual status in broiler soft tissues. Vet World.

[19] Sattar S, Hassan MM, Islam SKM, Alam M, Faruk MSA, Chowdhury S (2014). Antibiotic residues in broiler and layer meat in Chittagong district of Bangladesh. Vet World.

[20] Anika TT, Al Noman Z, Ferdous MRA, Khan SH, Mukta MA, Islam MS (2019). Time dependent screening of antibiotic residues in milk of antibiotics treated cows. J Adv Vet Anim Res.

[21] Cinquina AL, Longo F, Anastasi G, Giannetti L, Cozzani R (2003). Validation of a high-performance liquid chromatography method for the determination of oxytetracycline, tetracycline, chlortetracycline and doxycycline in bovine milk and muscle. J Chromat.

[22] Codex Alimentarius, International Food standard CAC/MRL 2-2015, CX/MRL 2, 2018. http://www.fao.org/fao-who-codexalimentarius/en.

[23] Chauhan SL, Priyanka GS, Jadhav VJ (2019). Determination of tetracycline residues in milk by high performance liquid chromatography. Int J Curr Microbiol Appl Sci.

[24] Verdon E, Couedor P, Roudaut B, Sandérs P (2005). Multiresidue method for simultaneous determination of ten quinolone antibacterial residues in multimatrix/multispecies animal tissues by liquid chromatography with fluorescence detection: Single laboratory validation study. J AOAC Int.

[25] Oluseyi T, Oyeyiola A, Rabiu B, Mbadiwe N, Olayinka K, Silva B (2016). Analysis of Antibiotics in poultry wastewater and droppings using solid phase extraction and High Performance Liquid Chromatography. J Chem Soc Nig..

[26] Harris DC (2012). Exploring chemical analysis.

[27] Fortt ZA, Cabello CF, Buschmann RA (2007). Residues of tetracycline and quinolones in wild fish living around a salmon aquaculture center in Chile. Rev Chil Infectol.

[28] Shim KB, Mok JS, Jo MR, Kim PH, Lee TS, Kim JH (2010). Residues of antibiotics in wild and cultured fishes collected from coast of Korea. Korean J Fish Aquat Sci.

[29] Barman A, Hossain M, Rahim M, Hassan M, Begum M (2018). Oxytetracycline residue in Tilapia. Bangladesh J Sci Ind Res.

[30] Barman AKA, Hossain MM, Rasul MG, Majumdar BC, Rahim MM (2018). Effects of oxytetracycline residues in Thai Koi (*Anabas testudineus* Bloch) collected from Sylhet, Bangladesh. Arch Agric Environ Sci.

[31] Sharafati-Chaleshtori R, Mardani G, Rafieian-Kopaei M, Sharafati-Chaleshtori A, Drees F (2013). Residues of oxytetracycline in cultured rainbow trout. Pak J Biol Sci.

[32] Barani A, Fallah AA (2014). Occurrence of tetracyclines, sulfonamides, fluoroquinolones and florfenicol in farmed rainbow trout in Iran. Food Agric Immunol.

[33] Turk E, Oguz H (2016). Investigation of tetracycline residues in fish caught from surrounding fish farms in Muğla district. Eurasian J Vet Sci.

[34] Baydan E, Kaya S, Çagirgan H, Yildirim E, Altintas L, Yurdakok B (2015). Investigation of some veterinary drug residues in sea water, sediment, and wild fishes captured around fish farms in the Aegean sea: oxytetracyline, ivermectin and emamectin. Ankara Üniv Vet Fak Derg.

[35] Mahmoudi R, Gajarbeygi P, Norian R, Farhoodi K (2015). Chloramphenicol, sulfonamide and tetracycline residues in cultured rainbow trout meat (*Oncorhynchus mykiss*). Bulg J Vet Med.

[36] Segmenoglu MS (2014). Searching some antibiotics that are tetracycline group in fish muscle tissue. AVKAE Magazine.

[37] Olatoye IO, Basiru A (2013). Antibiotic usage and oxytetracycline residue in African Catfish (*Clarias gariepinus* in Ibadan, Nigeria). World J Fish Mar Sci.

[38] Smith R, Donlon Coyne R, Cazabon DJ (1994). Fate of oxytetracycline in a freshwater fish farm: influence of effluent treatment system. Aquaculture.

[39] Kan CA, Meijer GLA (2007). The risk of contamination of food with toxic substances present in animal feed. Anim Feed Sci Technol.

[40] Victoria F, Samanidou NE (2007). Analytical strategies to determine antibiotic residues in fish. J Sep Sci.

[41] Mastovska K, Zweigenbaum J (2011). Multiresidue analysis of antibiotics in food of animal origin using liquid chromatography-mass spectrometry. Mass spectrometry in food safety: methods and protocols.

[42] Sapkota A, Sapkota AR, Kucharski M, Burke J, McKenzie S, Walker P (2008). Aquaculture practices and potential human health risks: current knowledge and future priorities. Environ Int.

[43] Heuer OE, Kruse H, Grave K, Collignon P, Karunasagar I, Angulo FJ (2009). Human health consequences of use of antimicrobial agents in aquaculture. Clin Infect Dis.

